# Does Industrial Air Pollution Increase Health Care Expenditure? Evidence From China

**DOI:** 10.3389/fpubh.2021.695664

**Published:** 2021-06-18

**Authors:** Jin-Sheng Shen, Qun Wang, Han-Pu Shen

**Affiliations:** ^1^School of Economics, Ocean University of China, Qingdao, China; ^2^Statistics and Data Science, Southern University of Science and Technology, Shenzhen, China

**Keywords:** industrial air pollution, health care expenditure, east, center, west, panel threshold regression model

## Abstract

This paper discusses the impact of air pollution on medical expenditure in eastern, central, and western China by applying the fixed-effect model, random-effect model, and panel threshold regression model. According to theoretical and empirical analyses, there are different relationships between the two indexes in different regions of China. For eastern and central regions, it is obvious that the more serious the air pollution is, the more medical expenses there are. However, there is a non-linear single threshold effect between air pollution and health care expenditure in the western region. When air pollution is lower than this value, there is a negative correlation between them. Conversely, the health care expenditure increases with the aggravation of air pollution, but the added value is not enough to make up for the health problems caused by air pollution. The empirical results are basically consistent with the theoretical analysis, which can provide enlightenment for the government to consider the role of air pollution in medical expenditure. Policymakers should arrange the medical budget reasonably, according to its situation, to make up for the loss caused by air pollution.

## Introduction

Industry has contributed a lot to economic development in any country. The rapid rise and vigorous development of industry is a vital consideration of economic development, which is reflected in the improvement of national income and per capita income, international trade, employment, urbanization level, and so on. The acceleration of industrialization is inevitable, but the continuous occurrence of natural and human disasters in recent years indicates that industry has become an important driver of environmental destruction and pollution (1). The reason why the manufacturing industry leads to air pollution is that they emit a lot of chemical gases such as nitrogen oxides and carbon oxides into the air, which not only aggravates air pollution but also harms people's health (2). Most of the pollution on earth can be attributed to industry (3), and industrial exhaust is the second-largest source of pollution after automobile exhaust (4). Due to the global environmental impact of industrial waste gas and detrimental effects for the health of humans, it has gradually become a topic of concern. This is mainly because industrial pollution is imposed on those who do not produce pollution in economic production in a negative external form. Air pollution is now the largest single environmental health risk on Earth and will soon become a vital cause of disease and death in low-income countries. These industrial exhaust emissions are often accompanied by excessive temperature and heat generation, which subtly changes the climate, changing people's arterial pressure and blood viscosity, and causes some cardiovascular and cerebrovascular diseases (5). The vigorous development of the economy has improved people's quality of life, but environmental problems are becoming increasingly prominent. Among 338 cities above prefecture level in China, only 99 cities meet environmental quality standards, accounting for only 29.3%, while the rest of the cities are heavily polluted areas, seriously exceeding air environmental quality standards (6). The health problems and related diseases caused by this pollution have been well-documented (7). For instance, industrial air pollution may cause carcinogenicity, pulmonary tuberculosis, pneumonia, cancer, and otitis media infection in early childhood (2, 8, 9). These diseases caused 9 million premature deaths worldwide in 2015, accounting for 16% of the deaths that year, three times the total number of deaths caused by AIDS, tuberculosis, and malaria, and 15 times the number of deaths caused by war and other violence.

The existence of air pollution exacerbates human health problems, which will lead to an increase in medical and health costs. The official statistics department of China shows that China's total health costs has risen from 2434.591 billion yuan in 2011 to 5259.828 billion yuan in 2017, an increase of more than two times (10). But the government's public health expenditure accounts for only about 30%, an increase of fewer than two times. It can be seen that the scale of public health expenditure in China is relatively small, and the increase in response to the deterioration of the health environment is insufficient. To a certain extent, it is not enough to make up for the negative influence of air pollution on residents' health (11). Therefore, it is particularly important to study the correlation between them, to provide a reference for a more reasonable arrangement of public health expenditure, and formulate more comprehensive environmental protection measures (12).

By combing the previous literature, we find that air pollution is related to the increase in health expenditure, and there is a non-linear relationship between them (13). However, previous papers on China's environmental pollution and medical expenditure regarded China as a whole, but few studies have focused on specific regions of China. As a country with obvious environmental problems, this topic is worth discussing. Therefore, the question remains as to what the relationship is between the two variables in different regions of China? Will industrial gas emissions increase medical expenditure? Is there a threshold between them? Will this relationship change with different resource endowments and geographical conditions in eastern China, central China, and western China?^1^ At present, the emission of sulfur dioxide, nitrogen oxides, and smoke (powder) dust is included in the industrial waste gas as the standard, which is used as the measurement indicator of air pollution degree. Based on the panel data of 14 years in China since 2002, this paper employs a fixed-effect model, random-effect model, and panel threshold regression model (PTRM) to explore the existence of threshold effect of industrial air pollution on medical expenses in eastern, central, and western China. From the results, we notice a positive correlation between air pollution and health care expenditure in the eastern and central regions, and the effect of the central region on air pollution is greater compared with the eastern region. In the western area, there is a non-linear threshold effect between the two. Air pollution reduces medical expenditure before the critical value but increases health care expenditure after the critical value. But the added value is not enough to bear the consequences of air pollution.

The contributions of this paper are as follows. First of all, previous studies have focused on the impact of air pollution on public health, but few studies have explored the impact of air pollution on medical expenditure. Although the existing literature has widely proved the relationship between the two, there has been no specific study on the three regions with different conditions in China. Therefore, we try to study the impact of air pollution on medical expenditure in the eastern, central, and western parts of China. Secondly, fine particulate matter (PM_2.5_) is often used as an important indicator of air pollution in the existing literature, and it is a single indicator. However, this paper uses industrial waste gas including carbon oxides, nitrogen oxides, and other gases as the environmental indicator, which can more fully reflect the impact of air pollution on medical expenditure.

In addition to the introduction, in the second part we comb the previous literature. The third part outlines the relevant theoretical model and the fourth part shows the research method used in this paper. The fifth part is the description of the data. The sixth part is the demonstration of the empirical results, and the seventh part is the conclusion.

## Literature Review

Studies have evidence that health care expenditure is associated with air pollution. A positive correlation between medical expenditure and atmospheric environment is observed (14, 15). According to World Trade Organization (WTO) estimates, the global economic cost of outdoor pollution will amount to 1% of the whole gross national product after about 40 years, and the related medical expenditure will dominate in the long run. Based on relevant data of 49 counties at a certain time point in Canada, it was found that counties with poor air quality had higher medical expenditure, while those with more investment in environmental quality had lower medical expenditure (11). Khoshnevis Yazdi et al. (16) studied the effect of environmental pollution on medical expenditure in Iran for 33 years since 1967. In the short term and long term, harmful gases such as sulfide and nitride did increase medical expenditure (16). The relationship between economic growth, environmental quality, and medical expenditure in the Middle East and North America from 1995 to 2014 was analyzed by Khoshnevis Yazdi and Khanalizadeh by the relative model (17). They concluded that carbon dioxide and PM_2.5_ emissions would have a significant positive impact on long-term health. Narayan P K and Narayan S used the panel data of Organization for Economic Co-operation and Development (OECD) countries from 1980 to 1999 to explore the short-term and long-term effects of environmental quality on health expenditure. The results showed that different air pollutants had significant positive effects on health expenditure in different periods (18). In addition, other studies on air pollution and mortality also indirectly illustrate this problem. Using panel data, Fotourehchi Z studied the effects of PM_10_ and CO_2_ on infant mortality and life expectancy at birth over 20 years since 1990 in several developing countries. It turns out that air pollution does increase mortality and reduce the benefits of health policies paying more attention to economic issues (19). Meanwhile, air pollution can aggravate respiratory diseases, especially in patients with asthma and chronic obstructive pulmonary disease (20).

However, not all conclusions are the same, some scholars in this field found that in the days after reaching high pollution levels (21, 22), the daily mortality rate will decrease. Arceo et al. concluded that environmental pollution and mortality are inversely correlated by verifying the data of typical developing countries (23). Based on the data of Ghana from 1970 to 2008, Micheal et al. applied FMOLS technology to study the long-term impact of carbon dioxide emissions and economic growth on medical expenditure. They confirmed that CO_2_ had no obvious effect on medical spending (24). More and more literature has discussed the impact of industrial pollution on health in China, including asthma, respiratory and other diseases related to death and the respiratory system (2, 25, 26). Medical expenditure accounts for a large part of China's total expenditure, which is the main economic burden of China. From the research of some scholars, it was found that provincial governmental health care spending per capita had a significant negative impact on industrial air pollution, which indicates that with the rise in the provincial governmental health care spending per capita, the awareness of environmental protection of people increases, and the industrial air pollution will be remarkably reduced (27). On the other hand, compared with developed areas, investment in less developed regions is more cost-effective, which can easier reduce air pollution (28). However, what is obvious is that few studies on the relationship between the two are found (11), of course, there are even fewer on how one party affects the other.

On the whole, existing research has discussed the impact of environmental pollution as a whole, but considering microdata to study the effect of environmental quality on individual medical spending and the differences in the impact on different groups and different regions has not been attempted. Understanding this problem is conducive to understanding the real economic pressure caused by environmental problems, and also helps local governments to formulate policies and targeted environmental protection measures according to regional differences. Given this, this paper describes the effect of air quality on individual medical spending and then analyzes the effect of air pollution on the individual medical expenditure of the provincial government based on China's provincial panel data. Because of the regional differences in air pollution sources in China, it is divided into eastern, central, and western China. Also, the threshold effect is studied in the areas where the relationship between them is not obvious.

## The Health Care Expenditure Model With Air Pollution

The theoretical model of the article draws on the analytical framework of Chang, Trivedi, Akpalu, and Normanyo (29, 30). It is assumed that the utility U of consumers decided by their physical condition h and their consumption of X of consumer goods. Consumers are rational and pursue utility maximization. Here, the utility function formula is as follows:

(1) $$\begin{array}{r} \text{u}=\text{u}\left(\text{x},\text{h}\right) \end{array}$$

where u_x_>0, u_h_>0, u_xh_=u_hx_>0, and u_xx_, u_hh_ <0, it is a little different from Chang and Trivedi. It is assumed that health status depends on I which is the level of individual investment in health, and I belong to derived demand. Due to the influence of a series of exogenous environmental factors, the return of I has both partial certainty and partial randomness. Because long-term exposure to air pollution particles will increase prevalence, the uncertainty of health status may come from the misdiagnosis, missed diagnosis, and disease recurrence of exposure to air pollution over a long time. The physical status of an individual is defined as follows:

(2) $$\begin{array}{r} \text{h}={\text{h}}_{0}+\text{eI} \end{array}$$

In Equation (2), h_0_ is the initial physical status of an individual, and e is the return (marginal rate of return) of the individual's medical investment (or medical expenditure). The price of goods x is standardized to 1, so the individual budget constraints are as follows:

(3) $$\begin{array}{r} \text{B}=\text{x}+\text{I} \end{array}$$

where B is the actual budget value, and the expected utility function of an individual is as follows:

(4) $$\begin{array}{r} \text{Eu}\left(\text{x},\text{h}\right)=\text{Eu}\left(\text{B}-\text{I},{\text{h}}_{0}+\text{eI}\right) \end{array}$$

It is assumed that the relative function about utility is additive for x and h, therefore we can get:

(5) $$\begin{array}{r} \text{u}\left(\text{x},\text{h}\right)=\text{u}\left(\text{x}\right)+\text{v}\left(\text{h}\right) \end{array}$$

At the same time, it is assumed that V (h) has the following special form:

(6) $$\begin{array}{r} \text{v}\left(\text{h}\right)=\text{ln}\left(\text{h}\right) \end{array}$$

Combining (5) and (6), formula (4) can be rewritten as follows:

(7) $$\begin{array}{r} Eu\left(x,h\right)=u\left(B-I\right)+E\left(\ln\left(h_{0}+eI\right)\right) \end{array}$$

Suppose that e obeys a 0-1 distribution with a mean value of μ, that is, μ = Ee. The formula (7) is as follows:

(8) $$\begin{array}{r} \text{Eu}\left(*\right)=\text{u}\left(\text{B}-\text{I}\right)+\text{μln}\left({\text{h}}_{0}+\text{I}\right)+\left(1-\mu \right)\text{ln}\left({\text{h}}_{0}\right) \end{array}$$

Then we obtain the first-order condition of the medical investment I:

(9) $$\begin{array}{r} -{\text{u}}_{\text{I}}\left(\text{B}-\text{I}\right)+\mu \frac{1}{{\text{h}}_{0}+\text{I}}=0 \end{array}$$

Equation (9) points out that under the equilibrium condition, the marginal return of investment in health (i.e., $\mu \frac{1}{{\text{h}}_{0}+I}$) should be equal to the cost of investing one yuan [i.e., *u*_*I*_(*B*−I)]. It can be seen that when other conditions remain unchanged, medical expenditure I decreases with the increase of initial health condition h_0_, and increases with the increase of budget constraint B and mean μ. The analysis means that, first of all, individuals with better initial health will spend less on health. Second, individuals with higher income will spend more. At the same time, a higher marginal rate of return μ will also promote medical expenses.

Finally, it is assumed that the random part of health status depends on the degree of exposure to the air of residents and other individual factors (A), so there are μ = μ(z; A). Among them, z is the external environment.

Suppose that the increase of external environmental pollution level leads to the increase of expected marginal return rate of medical expenditure, that is, u_z_ > 0. Furthermore, we can see that the medical expenditure I increases with the increase of environmental externality z. Suppose the general form of the investment equation of health care is as follows:

(10) $$\begin{array}{r} \text{I}=\text{f}\left({\text{h}}_{0},\text{B},\text{z};\text{A}\right) \end{array}$$

Equation (10) is the hedonic-type equation (1), where the medical expenditure depends on the environmental pollution and the individual characteristics of the residents.

## Methodology

### Panel Threshold Regression Model

The research method of this paper draws lessons from Hanson's threshold regression model (31). {*e*_*it*_, *AP*_*it*_, *x*_*it*_:1 ≤ *i* ≤ *n*, 1 ≤ *t* ≤ *T*}, we can establish the following model (32–34):

(11) $$e_{it}=\{\begin{array}{ll} \mu_{it}+\beta_{1}AP_{it}+\alpha_{1}^{\prime }x_{it}+\varepsilon_{it}, & if\;AP_{it}\leq \gamma \\ \mu_{it}+\beta_{2}AP_{it}+\alpha_{2}^{\prime }x_{it}+\varepsilon_{it}, & \;if\;AP_{it}>\gamma \end{array}$$

where *AP*_*it*_ is the provincial industrial waste gas discharge volume per capita, which is the central variable of the threshold model; γ is the relative value of *AP*_*it*_; *e*_*it*_ is the provincial health care expenditure per capita; β_1_ and β_2_ are the coefficients associated with *AP*_*it*_; *x*_*it*_ are other variables which can affect health care expenditure; α_1_ and α_2_ are relative coefficients of them; μ_*it*_ represents a fixed utility to adapt to the changes in conditions affecting different provinces; ε_*it*_ is random error term with $\varepsilon_{it}\sim \left(0,\sigma^{2}\right)$; and *i* and *t* represent provinces and time.

Equation (11) can be extended to the following forms:

(12) $$\begin{array}{l} e_{it}=\mu_{it}+\beta_{1}AP_{it}\psi \left(AP_{it}\leq \gamma \right)+\beta_{2}AP_{it}\psi \left(AP_{it}>\gamma \right)\\ \;+\alpha^{\prime }x_{it}+\varepsilon_{it} \end{array}$$

However, the actual situation may be different from the ideal state, and the number of turning points is uncertain. Hence, according to the reality, the threshold model can be expressed as (12):

(13) $$e_{it}=\{\begin{array}{ll} \mu_{it}+\beta_{1}AP_{it}+\alpha_{1}^{\prime }x_{it}+\varepsilon_{it}, & if\;AP_{it}\leq \gamma_{1}\\ \;\mu_{it}+\beta_{2}AP_{it}+\alpha_{2}^{\prime }x_{it}+\varepsilon_{it},\; & if\;\gamma_{1}<AP_{it}\leq \gamma_{2}\\ \mu_{it}+\beta_{3}AP_{it}+\alpha_{3}^{\prime }x_{it}+\varepsilon_{it}, & if\;AP_{it}>\gamma_{2} \end{array}$$

The regression shape of the above can also be shown as:

(14) $$\begin{array}{l} e_{it}=\mu_{it}+\beta_{1}AP_{it}\psi \left(AP_{it}\leq \gamma_{1}\right)+\beta_{2}AP_{it}\psi \left({\gamma_{1}<AP}_{it}\leq \gamma_{2}\right)\\ \;+\beta_{1}AP_{it}\psi \left(AP_{it}>\gamma_{2}\right)+\alpha^{\prime }x_{it}+\varepsilon_{it} \end{array}$$

where γ_1_ and γ_2_ are threshold values (γ_1_ < γ_2_). By analogy, multi-threshold models including three thresholds can be represented. In brief, the threshold regression model theory does not need to separate exogenous variables, so it does not need to give the specific form of the non-linear regression equation. The threshold and its estimated parameters are completely determined by the endogenous sample data (35–38).

## Data

This paper considers data from 2002 to 2015 including 31 provinces of China. Most of the air pollution data have been updated in recent years but the sample excludes years after 2015 considering the statistics of the data. The data sources are the China Environmental Yearbook, China Statistical Yearbook, and many other statistical yearbooks of China. According to geographical location and the level of economic development, China is divided into three parts: the east, the center, and the west. The eastern part refers to the provinces which first implemented the coastal open policy and a high level of economic development, the central part refers to less developed areas, and the western part refers to the least developed areas. Health care expenditure (HCE) is used to represent public health expenditure. Many previous studies use PM_2.5_ as an indicator of the severity of air pollution which can affect the health of the population, but industrial waste gas (IWG) is more comprehensive including nitrogen oxides, carbon oxides, and other gases. In this paper, industrial waste gas is used to measure industrial air pollution in every province and regarded as a threshold index. On the whole, air pollution will improve the prevalence of residents, and then expand medical expenditure, and a great many relative types of research have proved that the worse the air quality is, the more medical expenses there are. However, the characteristics of air pollution itself and the differences in different regions intensify the uncertainty, which leads to the study of the threshold effect of air quality.

In order to ensure the reliability of the empirical analysis, we also add four other control variables. The first is the aging process population (AP), with the development of the aging population, there are more diseases, such as hypertension, cancer, and so on, which are frequent diseases of the elderly, and the treatment of these diseases leads to the increase of medical expenditure of relevant departments. The second is education level (EL), which is obvious among the elderly. The improvement of education level directly promotes the increase of income, thus increasing medical expenditure. Moreover, the rise of education level will also improve the awareness of risk. People are willing to assume more resources to improve their health level, which will lead to an increase in medical expenditure. The third is income level (IL), which is regarded as the most significant determinant of consumer spending. Finally, hospital supply-beds (HSB) are usually used as a common measure of health care spending.

The original data are logarithmically processed to increase data stability and eliminate some metrological problems arising from the original data. Table 1 divides China into the east, center, and west for the mean, maximum, minimum, and std. dev of the data. What we can see in Table 1 is that the mean of health care expenditure in eastern China is higher. This may be related to the high medical level and advanced facilities in some eastern provinces. The industrial air pollution, proportion of the aging population, and education level are significantly higher than those of other areas of China. This is related to the large population and developed economy of eastern China. In terms of income level, western China is higher than the other two regions. This is related to the smaller population. When it comes to the hospital supply-beds, central China has more hospital supply-beds than the east and the west.

**Table 1:** Descriptive statistics of the variables.

		**Mean**	**Maximum**	**Minimum**	**Std. dev**.
East	HCE	5.65	7.63	3.60	0.98
	IWG	10.45	11.59	8.79	0.58
	AP	2.27	2.80	1.88	0.18
	EL	2.38	10.35	0.38	2.38
	IL	9.09	10.88	1.33	2.49
	HSB	1.32	1.92	0.76	0.35
Center	HCE	5.30	9.74	3.10	1.15
	IWG	10.15	14.76	8.73	0.77
	AP	2.01	2.55	0.62	0.42
	EL	1.60	2.96	0.74	0.52
	IL	8.23	10.35	0.67	2.95
	HSB	2.57	10.46	0.55	3.17
West	HCE	5.55	7.71	3.45	1.11
	IWG	10.09	12.46	6.10	1.23
	AP	2.08	2.65	1.56	0.23
	EL	2.34	4.00	0.86	0.66
	IL	9.48	10.33	8.69	0.45
	HSB	1.15	1.85	0.39	0.37

## Empirical Results

The first part uses fixed-effect and random-effect models to verify the impact of air pollution on medical expenditure in eastern, central, and western China. Model (1) and Model (2) are a fixed-effect model and random-effect model, respectively. Fixed-effect regression is a kind of variable method where changes are with individuals but not with time in control panel data. On the contrary, random-effect regression is a kind of variable method that changes with time but not with individuals in control panel data. The results are shown in Tables 2–4. Because the empirical results of the western region are not significant, and the coefficient of air pollution is different in the two models, therefore, the threshold effect of the western region is analyzed. In order to enhance the reliability of the results, two new variables are added. The results are shown in Tables 5–8.

**Table 2:** Results of the relationship between the two indexes in eastern China.

		**Variable**	**Estimated value**	**OLS se**	***t_***OLS***_***
East	Model (1)	IWG	0.2442***	0.0762	3.2047
		AP	0.0992	0.1402	0.7076
		EL	−0.0663	0.0699	−0.9458
		IL	1.5244***	0.0738	20.6558
		HSB	0.5321***	0.1121	4.7467
	Model (2)	IWG	0.2105***	0.0597	3.5260
		AP	0.0766	0.1299	0.5897
		EL	−0.0803	0.0669	−1.2003
		IL	1.5216***	0.0680	22.3765
		HSB	0.5666***	0.0934	6.0664

***, **, and *, respectively, indicate significance at the 1, 5, and 10% level.

The results in Table 2 show that the aggravation of air pollution has increased medical expenses in the east of China. The estimated coefficient of air pollution is 0.2442 and 0.2105, respectively when the significance level is 1% both in Model (1) and Model (2). In the meanwhile, what we can notice from Table 2 is that the impact of population aging and education level on health care expenditure in eastern China is not significant. However, the increase in income of people and the number of hospital beds will promote health care expenditure.

OECD (2016) has estimated, with the aggravation of environmental pollution, that economic costs will increase. Among the costs, medical expenditure will dominate for a very long time. According to Maryam Fattah, the deterioration of air quality will aggravate the amount of medical expenditure both of public and private health care expenditure in developing countries (39). With the increase of industrial emissions, the number of patients with related diseases will increase, so the government will invest more in health care expenditure (40). Eastern China has a developed economy and a high population density. On the one hand, the government has more funds to invest in medical expenditure. On the other hand, the particularity of air pollution compared with solid and liquid pollution has a greater impact on public health (41). For the control variable of income level, research shows that low-income people are more likely to give up medical care. Compared with the other two regions, the income per capita in eastern China is higher, and the increase of income per capita can promote the increase of medical expenditure (42). The supply cost of a hospital is the second-largest cost compared with wage cost, which accounts for 15% of the total hospital cost on average, and even up to 40% in some hospitals. The supply of beds is a part of the supply cost of the hospital, and its increase will naturally increase health care expenditure (43).

The results in Table 3 point out that there is a positive correlation between the two indexes in eastern China. The estimated coefficient of air environment is 0.9627 and 0.9117, respectively when the significance level is 1% both in Model (1) and Model (2). Income per capita and medical expenditure also show a significant positive impact, while the impact of hospital supply-beds on the central region is not significant. In addition, population aging is not significant in Model (1) but is positively correlated with medical expenditure in Model (2). Education level is negatively correlated with medical expenditure in Model (1), but not significantly correlated in Model (2).

**Table 3:** Results of the relationship between the two indexes in central China.

		**Variable**	**Estimated value**	**OLS se**	***t_***OLS***_***
Center	Model (1)	IWG	0.9627***	0.0321	29.9907
		AP	0.4378	0.3518	1.2445
		EL	−0.1648*	0.0824	−2.0000
		IL	1.0986***	0.2047	5.3669
		HSB	0.2183	0.1662	1.3135
	Model (2)	IWG	0.9117***	0.0389	23.4370
		AP	0.0573*	0.3344	1.1714
		EL	−0.1570	0.1009	−1.5560
		IL	1.1857***	0.1669	7.1043
		HSB	0.1309	0.1415	0.9251

*** and *, respectively, indicate significance at the 1 and 10% level.

Through the comparison of Tables 2, 3, we can conclude that the impact of air pollution on medical expenditure in central China is greater than that in eastern China. Due to the special characteristics of air pollution, the east and the center are adjacent, and air pollution in eastern China is more serious, which indirectly increases the air pollution in central China (44). Health care is necessary for the life of people (45). Different from the common necessities in economics, its value will increase with the improvement of people's health awareness and the development of society, so the corresponding medical expenditure will increase. In addition, the two indicators of population aging and education level may be influenced by time and individuals, and both models only fix one effect, which leads to unclear results of both control variables in eastern and central China.

Different from the former two regions in Table 4, the influence of air pollution on medical expenditure is not significant. We also find that the sign of coefficients in the two models is different. We speculate that the impact between the two variables in western China is non-linear, and there may be a certain threshold value. Therefore, the threshold test is carried out in the western region, and the results are displayed in Tables 5, 6.

**Table 4:** Results of the relationship between the two indexes in western China.

		**Variable**	**Estimated value**	**OLS se**	***t_***OLS***_***
West	Model (1)	IWG	0.0584	0.0491	1.1894
		AP	0.5237	0.2931	1.7868
		EL	−0.4407***	0.0607	−7.2603
		IL	1.7290***	0.2090	8.2727
		HSB	0.2285	0.1832	1.2473
	Model (2)	IWG	−0.1071	0.0999	−1.0721
		AP	−0.7252***	0.1874	−3.8698
		EL	−0.0642	0.0634	−1.0126
		IL	2.3486***	0.1955	12.0133
		HSB	0.2516	0.1649	1.5258

***, **, and *, respectively, indicate significance at the 1, 5, and 10% level.

**Table 5:** Results of threshold effects between air pollution and health care expenditure.

	**Single threshold effect test**			**Double threshold effect test**			
	**Threshold value**	***F*-statistics**	***p*-value**	**Threshold value**		***F*-statistics**	***p*-value**
West	9.6785*	27.0841	0.0900	9.6785	11.1714	5.3407	0.78

*, respectively, indicate significance at 10% level.

**Table 6:** Estimated coefficients of air pollution and health care expenditure.

**Region**	**Coefficient**	**Estimated value**	**OLS se**	***tOLS***	**White se**	***t_***White***_***
West	${\hat{\beta }}_{1}$	−0.0180	0.0606	−0.2970**	0.0475	−0.3789***
	${\hat{\beta }}_{2}$	0.0094	0.0596	0.1577	0.0471	0.1996**

*OLS se (White se) refers to homogeneous (heterogeneous) standard deviations*.

*** and **, respectively, indicate significance at the 5 and 10% level.

In western regions, the relationship between the two variables is in a U-shape, i.e., heavier air pollution improves the health care expenditure. In Table 5, when the industrial air pollution exceeds the turning point of 9.6785, the coefficient of air pollution changes to 0.0094, i.e., excessive industrial air pollution increases medical expenditure.

The relationship between the two variables is easy to see. With the aggravation of pollution, medical expenditure also increases when industrial air pollution is more than the inflection point, which is connected with the economic growth of the western regions. With the improvement of the economic level, air pollution has caused a huge loss on China's economy including health care expenditure (46). The economic development of western China is slow, the development policy of western China since 2000 has been implemented to balance the economy between western and eastern China. Traditional industries have been moved from the east to west (47). Health care expenditure has been added with the improvement of the economy in western China. In order to control irrational increases in medical expenditures, the Chinese government has initiated a new health reform since 2009 (48). Meanwhile, public awareness of air quality in western China is increasing (49). But the emergence of the inflection point shows some problems in the relationship between health care expenditure and air pollution. The influence between the two variables is decreasing. The aggravation of air pollution has an impact on health expenditure through various channels. On the one hand, air pollution increases health expenditure according to its diffusibility in different areas; on the other hand, economic growth may reduce health expenditure by the improvement of medical technology and increasing the employment rate, resulting in a smaller coefficient (50). In general, the added value of health care expenditure is too small to make up for the health loss caused by air pollution.

As is revealed in Table 7, population aging contributes to more health care expenditure in western China. Research from Milena et al. shows that the increasing number of the elderly leads to more payments on medical health compared with America (12). The population aging aggravates the long-term care expenditure to a great extent, and more attention should be paid to the medical care system (51). Conversely, there is a negative relationship between the provincial income per capita and expenses on health care. Increasing income level has reduced the development of medical services. In fact, health care is very necessary for the life of people. Thus, people will pay less on the expenses of health care according to the characteristics of necessities. But the size of income elasticity varies from country to country, with poorer countries having a higher elasticity (52). Finally, needing more hospital supply-beds also increases health care expenditure. Hospital supply-beds sometimes reflect the surge capacity, the increase of hospital supply-beds means that something associated with it also increases, leading to more health care expenditure (53).

**Table 7:** Estimated coefficients of the control variables.

**Region**	**Cofficient**	**Estimated value**	**OLS se**	***t_***OLS***_***	**White se**	***t_***White***_***
West	${\hat{\alpha }}_{1}$	0.6607	0.2177	3.0349***	0.2226	2.9681***
	${\hat{\alpha }}_{2}$	−0.2768	0.0860	−3.2186***	0.0821	−3.3715***
	${\hat{\alpha }}_{3}$	−0.2938	0.1280	−2.2953**	0.1199	−2.4504
	${\hat{\alpha }}_{4}$	2.0857	0.1664	12.5343***	0.1773	11.7637***

OLS se (White se) refers to homogeneous (heterogeneous) standard deviations. The estimated coefficients of ${\hat{\alpha }}_{\text{1}}$ is for the aging process of population, ${\hat{\alpha }}_{\text{2}}$ is for education level, ${\hat{\alpha }}_{\text{3}}$ is for income level, and ${\hat{\alpha }}_{\text{3}}$ is for hospital supply-beds. *** and **, respectively, indicate significance at the 5 and 10% level.

In order to enhance the reliability of the conclusion, another two new control variables are selected for the robustness test. The new control variable includes the urbanization level, which refers to the provincial proportion of the urban population to the total population. It evidences that the acceleration of urbanization means more economic activities that contribute to the development of the economy in some city and country (54). Another control variable is hospital supply-doctors, an effective measure of the medical level, which is the number of technicians per 1,000 people in every province of China (55). The two variables are included in the following threshold model of (15) and (16).

(14) $$\begin{array}{l} Model\left(1\right):e_{it}=\mu_{it}+\beta_{1}AP_{it}\left({AP}_{it}\leq \gamma \right)+\beta_{2}AP_{it}\\ \;\left({AP}_{it}>\gamma \right)+\alpha_{1}^{{\;}^{\prime }}HCE_{it}+\alpha_{2}^{{\;}^{\prime }}IWG_{it}+\alpha_{3}^{{\;}^{\prime }}EL_{it}\\ \;+\alpha_{4}^{{\;}^{\prime }}IL_{it}+\alpha_{5}^{{\;}^{\prime }}HSD_{it} \end{array}$$

(14) $$\begin{array}{l} Model\left(2\right):e_{it}=\mu_{it}+\beta_{1}AP_{it}\left(AP_{it}\leq \gamma \right)+\beta_{2}{AP}_{it}\left({AP}_{it}>\gamma \right)\\ \;+\alpha_{1}^{{\;}^{\prime }}HCE_{it}+\alpha_{2}^{{\;}^{\prime }}IWG_{it}+\alpha_{3}^{{\;}^{\prime }}EL_{it}+\alpha_{4}^{{\;}^{\prime }}IL_{it}+\alpha_{5}^{{\;}^{\prime }}HSD_{it}\\ \;+\alpha_{6}^{{\;}^{\prime }}UL_{it} \end{array}$$

Table 8 indicates that there is still a single threshold effect in western China for different empirical models. The robustness test shows that the previous test between the two indexes is obvious in the western region even if different control variables are added. These results are consistent with those in Table 5 and the conclusions obtained are more reliable.

**Table 8:** Results of threshold effects between air pollution and health care expenditure.

		**Single threshold effect test**			**Double threshold effect test**			
		**Threshold value**	***F*-statistics**	***p*-value**	**Threshold value**		***F*-statistics**	***p*-value**
West	Model (1)	9.6041**	24.4703	0.0415	9.6041	11.1714	6.3686	0.1600
	Model (2)	9.6035*	27.7899	0.0800	0.0636	11.1708	7.0262	0.1700

** and *, respectively, indicate significance at the 5 and 10% level.

The continuous growth of China's economy is accompanied by a large amount of coal and oil burning, which has caused a great amount of air pollution and seriously affected the public health level, resulting in the asymmetry between medical expenditure and air pollution. However, the relevant literature studies China as a whole and the conclusions are not convincing enough. Therefore, this paper considers the fixed-effect model, random-effect model, and PTRM to confirm the relationship between the two variables in eastern, central, and western China to provide more convincing conclusions. The results indicate that there is a linear positive correlation between them in eastern China and central China, but in western China, where the correlation between the two is non-linear and asymmetric, producing a critical industrial emission per capita value. When air pollution is lower than the critical value, it is negatively correlated with health care expenditure. On the contrary, when air pollution is higher than the critical value, the aggravation of air pollution will increase the health care expenditure, but the increase is not obvious. Therefore, the corresponding provinces of each region should reasonably arrange the medical expenditure budget, to make up for the health problems caused by the air environment.

## Conclusions

Through the panel data of 14 years in China since 2002, this paper employs a fixed-effect model, random-effect model, and PTRM to explore the existence of the threshold effect of industrial air pollution on medical expenses in eastern, central, and western China. In this study, industrial air pollution is defined as the provincial industrial emissions per capita. The results indicate that serious air pollution promotes the increase of medical expenditure in the eastern and central regions, and the impact of the central region on air pollution is greater than that of the eastern region. In the west of China, there is a non-linear threshold effect between the two. Air pollution reduces the expenses of health care before the critical value but increases health care expenditure after the critical value. But the added value is not enough to bear the consequences of air pollution. These conclusions provide valuable insights for government departments to arrange the health care expenditure budget in light of the increasingly serious air pollution problem. Air pollution will increase medical costs, so the government should reasonably arrange the scale of health expenditure to make up for the negative impact of air pollution. The correlation between air pollution and health care expenditure could be a fruitful area for future study, which can be used for reference by other developing countries. We intend to examine the relationship between the level of economic development in different regions of China and the incidence of infectious diseases in future studies; thus, a new method.

## Data Availability Statement

The datasets presented in this study can be found in online repositories. The names of the repository/repositories and accession number(s) can be found in the article/supplementary material.

## Author Contributions

H-PS: conceptualization, methodology, and software. J-SS: visualization and investigation. QW: data curation, writing—reviewing, and editing. All authors contributed to the article and approved the submitted version.

## Conflict of Interest

The authors declare that the research was conducted in the absence of any commercial or financial relationships that could be construed as a potential conflict of interest.

## Footnotes

^1^Eastern China includes: Liaoning province, Beijing, Tianjin, Hebei province, Shanghai, Jiangsu province, Zhejiang province, Fujian province, Shandong province, Guangdong province, Hainan province. Central China includes: Jilin province, Heilongjiang province, Shanxi province, Anhui province, Jiangxi province, Henan province, Hubei province, Hunan province. Western China includes: Neimenggu, Guangxi, Chongqing, Sichuan province, Guizhou province, Yunnan province, Xizang, Shanxi province, Gansu province, Qinghai, Ningxia, Xinjiang.
